# The relationship between moral distress and clinical care quality among nurses: an analytical cross-sectional study

**DOI:** 10.1186/s12912-024-02368-z

**Published:** 2024-10-09

**Authors:** Fateme Safari, Ali MohammadPour, Mahdi BasiriMoghadam, Alireza NamaeiQasemnia

**Affiliations:** Department of Medical Surgical Nursing, School of Nursing, Nursing Research Center, Gonabad University of Medical Sciences, Gonabad, Iran

**Keywords:** Moral Distress, Clinical Care Quality, Nurses

## Abstract

**Background:**

Nurses constitute the largest group of service providers in the healthcare system and significantly influence the quality of healthcare services. Factors such as ethical considerations may be related to the quality of care. This study aimed to determine the relationship between moral distress and the quality of clinical care among nurses working in Gonabad, Iran.

**Methods:**

An analytical cross-sectional study was conducted on 252 nurses working in emergency, internal medicine, surgery, psychiatry, critical care and maternity wards at Allameh Bohlool Hospital from May to July 2023. This research used demographic information questionnaire, the revised Moral Distress Scale (MDS-R), and the Quality Patient Care Scale (QUALPAC). The significance level for the study was set at *p* < 0.05.

**Results:**

There was a significant relationship between the frequency of moral distress and the quality of clinical care (*p* = 0.032), as well as between the intensity of moral distress and the quality of clinical care (*p* = 0.043). Nurses who experienced moral distress more frequently and intensely provided better quality care. However, there was no significant relationship between the effect of moral distress and the quality of clinical care (*r* = 0.032, *p* = 0.619). Additionally, a significant statistical relationship was found between the intensity of moral distress and the physical dimension of clinical care quality (*r* = 0.171, *p* = 0.007), indicating that increased moral distress intensity was associated with higher quality of physical care.

**Conclusions:**

Nurses who experience higher levels of moral distress, both in terms of frequency and intensity, perform better in the care they provide and deliver it in the best possible manner, particularly in the physical dimension of care.

## Introduction

Nurses are the largest group of service providers in the healthcare system and significantly impact the quality of healthcare services. Factors such as ethical considerations can influence the quality of care [1]. Nursing uses the concept of ethics to standardize and hold accountability in care [2]. The advancement of knowledge and technology has led to a focus on ethical issues in various professions, especially nursing. Nurses constantly face ethical dilemmas in their work environment, affecting all professional aspects [3]. According to Jameton, three experiences related to ethical problems that a nurse may encounter are moral uncertainty, ethical dilemmas, and moral distress [4].

Studies indicate that nurses working in developing countries report higher levels of moral distress compared to those in developed countries [5]. Moral distress, introduced by Jameton, occurs when a person is in a situation completely against their moral beliefs and, despite moral reasoning, cannot perform the ethical action due to real or perceived barriers [6]. Moral distress is influenced by environmental, occupational, organizational, and individual factors and has ambiguous consequences [7, 8]. Epstein and Hamric described moral residue as the lasting impact felt after a morally troubling situation. Unresolved moral distress can accumulate over time, easily growing. When a nurse repeatedly experiences moral distress, the starting point for moral distress becomes higher due to accumulated moral residue. The more frequently moral distress is experienced, the more intense it becomes, known as the “Crescendo Effect” [9]. Any ethical incident, even if it occurs once with any intensity, will have acute effects on the individual; repeated occurrences lead to chronic effects [10]. Moral distress can result in negative outcomes such as headaches, insomnia, hopelessness, withdrawal from family, and fear of going to work [11, 12]. When nurses lack the skills to successfully cope with moral distress, they may disengage morally and experience moral non-participation [13].

Positive outcomes have also been reported for moral distress. Diverse experiences in dealing with distressing conditions play a significant role in professional development and, by promoting positive values, improve patient care [14]. In a study by Wiegand and Funk, nurses noted changes after experiencing moral distress, including quicker interventions, enhanced family support, assertiveness, and consulting ethics committees. These experiences motivated them to intervene in similar situations in the future, suggesting that moral distress can catalyze positive change among nurses [12]. Traditional views of moral distress limit ethical analysis, assuming the distressed individual has already made a moral judgment. However, moral distress can prompt deeper inquiry, encouraging reflection, information gathering, and questioning of ethical judgments, empowering nurses to exercise their moral agency [15]. How nurses cope with moral distress may impact their clinical performance and personal lives, though there is some doubt about this [16].

Care is a fundamental component of healthcare services. Among the various types of care provided in healthcare settings, nursing care holds particular importance, and providing quality care is a priority in the healthcare system. In many countries, hospital accreditation is influenced by the quality of nursing care [17, 18]. Meeting the individual needs of patients is the core of nursing care, and the ultimate goal of nurses is to provide quality patient care [17, 19]. Today, efforts to improve quality and evaluate this variable in nursing systems are being emphasized [20]. Quality healthcare services mean achieving the most desirable health outcomes, where the services provided are effective, efficient, and cost-effective [21, 22]. Nurses are legally and ethically accountable for the quality of care they provide, making their perspective on defining healthcare quality particularly significant. Healthcare providers define quality as “doing the right thing, at the right time, and doing it right the first time,” but patients often define quality based on what personally matters to them [23].

Moral distress may lead to a reduction in care quality, which can further cause a conflict of conscience and result in moral distress [24]. Few studies have examined the relationship between moral distress and the quality of clinical care among nurses. These studies have reported varying results, with some finding a relationship between moral distress and clinical care quality [25] and others finding no such relationship [26]. This study aimed to determine the relationship between moral distress and the quality of clinical care among nurses.

## Methods

### Study design

An analytical cross-sectional study was conducted in Gonabad, northeast of Iran from May to July 2023.

### Setting and sample

The target group consisted of nurses working in the public sector of Gonabad County. The sample size was determined using G-power software version 7.9.1.3 and the Correlation: Bivariate normal model test from the Exact distribution family, with a correlation coefficient of -0.18 between the two quantitative variables of clinical care quality and moral distress. This calculation resulted in a required sample size of 239, which was adjusted to 250 to account for a 5% attrition rate. The correlation coefficient (r) of -0.18 was based on a similar study [25]. The 252 participating nurses were required to have at least a bachelor’s degree in nursing, a minimum of six months of work experience in hospital wards, current clinical employment, no history of severe stress based on self-report, and consent to participate in the study.

### Data collection

After the study protocol was approved by Gonabad University of Medical Sciences and the ethics committee granted approval, an introduction letter was obtained from the Vice-Chancellor for Research and Technology of the university and submitted to Allameh Bohlool Hospital. Permission to access clinical wards was granted by the hospital’s research department, and introduction letters were sent to head nurses through the administrative system. After which the researcher visited the head nurse at Allameh Bohlool Gonabadi Hospital to compile a confidential list of the hospital’s employed nurses, ensuring non-disclosure of information. A total of 260 nurses met the inclusion criteria and were selected via census sampling. Allameh Bohlool Gonabadi Hospital is a public teaching hospital with seven floors and 278 active beds. It comprises emergency departments, three surgical wards, two internal medicine wards, a psychiatric ward, three intensive care units (cardiac, neonatal, and general), and obstetrics and gynecology wards.

The researcher visited the nurses during morning, evening, and night shifts, typically in the middle of the shift. After verifying the inclusion criteria, explaining the study, assuring confidentiality of the information, clarifying that names and surnames were not required, and obtaining written consent, the targeted questionnaires were distributed and completed. Completing the questionnaire took approximately 15–20 min. Given the nurses’ busy schedules, they were allowed to specify a convenient time for returning the questionnaires, which were then collected. A designated location in each ward was arranged in coordination with the head nurse for nurses to place their completed questionnaires if they chose to do so. Participating nurses were informed that their participation or non-participation would not affect their job performance evaluations. Data collection occurred over two months. Data were collected directly by the researcher, who approached the nurses, obtained written consent, and then administered the questionnaires.

### Instruments

Three questionnaires were utilized in this study:

1. **Demographic Information Form**: This form gathered information on age, gender, marital status, number of children, education, employment status, work experience, shift work, number of night shifts and working hours per month, ward, economic status, executive position, and training in ethics.
2. **Moral Distress Scale-Revised (MDS-R)**: Originally developed by Jameton in 1984 and later revised by Corley and Hamric, this tool measures three dimensions of moral distress: frequency, intensity, and impact [27]. The MDS-R consists of 21 items rated on a five-point Likert scale, where frequency is scored from ‘never’ (0) to ‘daily’ [4] and intensity from ‘none’ (0) to ‘very high’ [4]. The impact of moral distress for each item is calculated by multiplying the intensity score by the frequency score, resulting in item scores ranging from 0 to 16. The total score of moral distress ranges from 0 to 336, with higher scores indicating greater moral distress. The tool also includes an open-ended question about other situations causing moral distress and two closed-ended questions about previous decisions to leave clinical practice or the nursing profession due to moral distress. These three questions do not contribute to the overall moral distress score. The internal consistency of this tool was confirmed with a Cronbach’s alpha of 0.89 in the nursing population. In Iran, it has been psychometrically validated. For instance, in the study by Mahdavi Feshtami et al. (2016), the internal consistency was 0.84 for frequency, 0.82 for intensity, and 0.86 for the overall moral distress score, with an overall Cronbach’s alpha of 0.86 [28]. Another tool, the Measure of Moral Distress for Healthcare Professionals (MMD-HP), developed in 2019 to assess the root causes of moral distress among healthcare professionals, requires further validation and has not yet been psychometrically tested in Iran. Hence, the Persian version of MDS-R was employed in this study [29].
3. **Quality Patient Care Scale (QUALPAC)**: This scale was developed by Wandelt in 1972 and has been used in the USA, UK, and Nigeria [23]. It assesses the quality of clinical care from the perspectives of nurses and patients [30]. The original questionnaire contained 68 items, which were culturally adapted and expanded to 72 items by Khoshkho in 2004 in Tabriz, Iran. The QUALPAC measures nursing care quality across three dimensions: psychosocial (33 items), physical (26 items), and communication (13 items). Each item is rated on a five-point Likert scale with options ranging from ‘not applicable’ to ‘always’. The quality of nursing care is scored as undesirable (0 to 1.89), somewhat desirable (1.90 to 2.63), and desirable (2.64 to 4). The reliability of the questionnaire was confirmed in a study by Khaki et al., where it was completed by 20 nurses, resulting in a Cronbach’s alpha of 0.96 [21].

### Data analysis

Data analysis was performed using SPSS software version 19. Descriptive statistics were used to summarize the demographic data, moral distress, and clinical care quality variables, including absolute numbers (n), prevalence (%), and measures of central tendency and dispersion (e.g., mean and standard deviation) as appropriate for the data type. For inferential statistics, the normality of data distribution was assessed using the Kolmogorov-Smirnov test. Due to the non-establishment of Cochran’s condition, the Exact *p*-value was reported for determining the relationship between the frequency and intensity of moral distress and clinical care quality using the chi-square test. The Spearman’s rank correlation was employed to assess the relationship between the impact of moral distress and clinical care quality. Results were considered significant at a *p*-value of less than 0.05.

### Ethical considerations

The study protocol was approved by the ethics committee of Gonabad University of Medical Sciences (Ethics Code: IR.GMU.REC.1402.015). All ethical guidelines were strictly followed, and participants were assured that their data would be kept confidential.

## Results

### Characteristics of the participants

Among the total 252 nurse participants, the majority were female, young, married, had 1–2 children, held a bachelor’s degree in nursing, were officially employed, had less than 10 years of work experience, and worked rotating shifts (Table 1).

**Table 1:** Demographic characteristics of the participants (*N* = 252)

Variable	Categories	*N* (%)
Gender	Male Female	161 (64.1) 90 (35.9)
Age(years)	20–30 30–40 40–50 50–60	108 (43.7) 99 (40.1) 34 (13.8) 6 (2.4)
Material status	Single Married	41 (16.3) 211 (83.7)
Child (person)	0 1–2 2–4	83 (35.6) 129 (55.4) 21 (9.0)
Education	BSc MSc	184 (74.8) 62 (25.2)
Employment Status	Plan Contract Formal	30 (15.4) 28 (14.3) 137 (70.3)
Work Experience(years)	< 10 10–20 20–30	155 (62.8) 70 (28.3) 22 (8.9)
Shift work	Fixed Rotation	32 (13.2) 210 (86.8)
Number of night shifts per month	0–4 4–8 8–12	60 (24.3) 173 (70.0) 14 (5.7)
working hours per month	60–120 120–180 180–240 240–300	6 (2.7) 105 (46.9) 94 (41.9) 19 (8.5)
Ward	Intensive General	120 (50.8) 116 (49.2)
Income status	Less than living expenses Equal living expenses More than living expenses	42 (18.9) 174 78.4) 6 (2.7)
Training in ethics	Yes No	166 (74.4) 57 (25.6)
Experience or having an executive position	Yes No	39 (15.5) 213 (84.5)

### Frequency and intensity of moral distress

About 51.2% of the nurses reported a moderate frequency of moral distress, with a mean score of 1.56 (SD = 0.70). Additionally, 39.3% of the nurses reported a moderate intensity of moral distress, with a mean score of 1.93 (SD = 0.87). Furthermore, 57.9% of the participants reported a low impact of moral distress, with a mean score of 1.5 (SD = 0.64) (Table 2).

**Table 2:** Frequency distribution of research participants according to dimensions of moral distress

Variable	Low *N* (%)	moderate *N* (%)	High *N* (%)	Very high *N* (%)	Mean (SD)
Frequency of moral Distress	59 (23.4)	129 (51.2)	56 (22.2)	8 (3.2)	1.56 (0.70)
Intensity of moral Distress	38 (15.1)	99 (39.3)	80 (31.7)	35 (13.9)	1.93 (0.87)
Impact of moral distress	146 (57.9)	86 (34.1)	20 (7.9)	35 (13.9)	1.5 (0.64)

The highest mean scores in the subscale of the impact of moral distress were associated with “carrying out unnecessary physician orders” and “performing life-saving actions that merely delay patient death.”

### Quality of clinical care

Approximately 87.6% of the nurses rated the quality of clinical care as desirable, with a mean score of 3.12 (SD = 0.46). Additionally, 82.3% rated the quality of clinical care in the psychosocial dimension as desirable, 86.5% in the physical dimension, and 88.5% in the communication dimension (Table 3).

**Table 3:** Frequency distribution of research participants according to Quality of Clinical Care dimensions

Variable	Undesirable *N* (%)	Relatively Desirable *N* (%)	Desirable *N* (%)	Mean (SD)
Quality of total clinical care	5 (2)	26 (10.4)	219 (87.6)	3.12 (0.46)
Psychosocial dimension	7 (2.8)	37 (14.9)	204 (82.3)	3 (0.51)
Physical dimension	7 (2.8)	27 (10.7)	218 (86.5)	3.20 (0.52)
Communication dimension	3 (1.2)	26 (10.3)	223 (88.5)	3.23 (0.56)

### Relationship between moral distress and quality of clinical care

There was a significant direct relationship between the frequency of moral distress and the quality of clinical care (*p* = 0.032), as well as between the intensity of moral distress and the quality of clinical care (*p* = 0.043). However, there was no significant relationship between the impact of moral distress and the quality of clinical care (*r* = 0.032, *p* = 0.619). Additionally, there was a statistically significant relationship between the intensity of moral distress and the physical dimension of the quality of clinical care (*r* = 0.1, *p* = 0.007) (Tables 4, 5 and 6).

**Table 4:** The relationship between the frequency and intensity of moral distress with the quality of total clinical care

Variable		Quality of total clinical care			χ2 result
		Undesirable *N* (%)	Relatively Desirable *N* (%)	Desirable *N* (%)	
Frequency of moral Distress	Low	4 (6.9)	4 (6.9)	50 (86.2)	Exact *p* = 0.032
	Moderate	1 (0.8)	17 (13.2)	111 (86.0)	
	High	0 (0.0)	3 (5.5)	52 (94.5)	
	Very high	0 (0.0)	2 (25.0)	6 (75.0)	
Intensity of moral Distress	Low	3 (8.1)	3 (8.1)	31 (38.8)	Exact *p* = 0.043
	Moderate	1 (1.0)	10 (10.1)	88 (88.9)	
	High	1 (1.3)	12 (15.2)	66 (83.5)	
	Very high	0 (0.0)	1 (2.9)	34(97.1)	

**Table 5:** Relationship between the effect of moral distress and the quality of clinical care of nurses

Variable	Mean (SD)	Spearman’s test result
impact of moral distress	81.47 (53.71)	*r* = 0.032
Quality of total clinical care	3.12 (0.47)	*p* = 0.619

**Table 6:** Relationship between moral distress dimensions and quality of clinical care dimensions

Variable	dimensions of moral distress		
Quality of Clinical Care dimensions	Frequency of moral Distress	Intensity of moral Distress	Impact of moral distress
Psychosocial dimension	*r* = 0.03^*^ *p* = 0.678	*r* = 0.10^*^ *p* = 0.115	*r* = 0.03^*^ *P* = 0.678
Physical dimension	*r* = 0.3^*^ *P* = 0.575	*r* = 0.171^*^ *P* = 0.007	*r* = 0.3^*^ *P* = 0.575
Communication dimension	*r* = -0.06^**^ *p* = 0.641	*r* = 0.05^**^ *P* = 0.355	*r* = -0.09^**^ *P* = 0.891

*Pearson test **Spearman’s test

## Discussion

This study examined the relationship between moral distress and the quality of clinical care provided by nurses. The findings revealed a significant relationship between the frequency and intensity of moral distress and the quality of care, with higher distress levels correlating with better care. However, there was no significant link between the impact of moral distress and care quality. Additionally, a significant relationship was found between the intensity of moral distress and the physical dimension of care quality, showing that greater distress intensity was associated with improved physical care.

In alignment with the present study, Yu et al.‘s research on Chinese emergency nurses found that moral courage and educational workshops can reduce moral distress and improve nurses’ social performance [31]. Mert Boğa et al.‘s study in Turkey demonstrated that with increased ethical sensitivity, nurses’ perception of care quality improves, highlighting ethical sensitivity as a crucial factor in nursing care quality [2]. Ohnishi et al., in their study on psychiatric nurses in Finland and Japan, found a general relationship between moral distress and ethical sensitivity, with nurses possessing higher ethical sensitivity experiencing greater moral distress [32]. Khodavisi et al.‘s research in western Iran reported a direct and strong correlation between ethical sensitivity and safe nursing care [33]. Khorani et al. identified ethical sensitivity among nurses in Qazvin hospitals as a factor enhancing adherence to ethical principles and encouraging higher quality care. High levels of moral distress during nursing care necessitate ethical decision-making, where higher ethical sensitivity can significantly enhance nurses’ ability to make ethical decisions during care. A lack of ethical sensitivity or inability to identify and address ethical challenges may lead to suboptimal care behaviors [34]. Heydari et al. found a direct and significant relationship between the quality of nursing care and moral intelligence, defining moral intelligence as the recognition of right from wrong, guiding other forms of intelligence towards valuable actions [35].

According to Haahr et al., besides individual nurse competencies, organizational structures, policy programs, and hospital cultures influence nurses’ actions based on ethical beliefs and professional ethics [36]. Deschenes et al. highlighted the complexity of moral distress in nursing, noting its multifaceted nature. They emphasized that while external constraints such as institutional and systemic limitations contribute significantly to moral distress, internal factors like psychological imbalance and painful emotions also play a role. They proposed replacing the term “internal constraints” with “internal characteristics” to shift the focus from individual culpability to organizational responsibility, highlighting the need for systemic change. Moral distress can have detrimental effects, including blaming others, self-blame, depression, and anxiety, which may lead to burnout and compromise patient care. Conversely, resolving moral distress can lead to personal and professional growth, enhancing nurses’ ethical sensitivity and care skills [37].

Jansen et al. further explored the link between moral distress, conscience, and the quality of nursing care. They emphasized how moral distress can compromise care quality, leading to a troubled conscience. Conscience acts as a motivator for ethical reflection and can expose unethical practices, but prolonged moral distress can lead to negative physical and psychological consequences such as fatigue and insomnia. While intermittent moral distress can foster ethical reflection and alertness to ethical dilemmas, its chronic presence can result in frustration and mental and physical health issues [24].

The findings suggest that ethical sensitivity may mediate the relationship between moral distress and clinical care quality among nurses. Morley and Sankary’s study emphasized moral distress as a signal of ethical issues, prompting actions to address them [38]. However, studies such as those by Azarmi et al., Moghaddam et al., and Amiri et al. present conflicting results regarding the impact of moral distress on care quality [26, 39, 40].

Moral distress, if unresolved, can lead to moral numbness and burnout among nurses [41]. DeKeyser Ganz and Berkovitz found an inverse relationship between moral distress frequency and care quality, indicating that increased distress correlates with decreased care quality [25]. Situations causing moral distress demand nurses’ time and attention, potentially leading to inadequate care and decreased safety [39].

Despite these challenges, moral distress can foster autonomy and professional development. Nurses may develop strategies to cope with distress and engage in reflective practice [42]. Studies by Mahdavi Feshtami et al., Mohammadi et al., and Rahmanian et al. reported moderate levels of moral distress, while Shafiee et al. found low distress levels among hospital nurses in Bushehr [6, 28, 43, 44].

However, disparities exist in the reported frequency and intensity of moral distress across studies. Factors may occur frequently but with low intensity or vice versa. For example, Sauerland et al. reported low frequency but moderate intensity of moral distress in an American teaching hospital [27].

In summary, moral distress poses challenges to care quality but also presents opportunities for professional growth. Understanding its nuances is crucial for addressing its impact on nurses and patient care.

In conclusion, ethical sensitivity emerges as a critical factor that can mediate the relationship between moral distress and clinical care quality. While moral distress often poses challenges, it also has the potential to drive nurses towards higher quality care if managed effectively. Organizational support, ethical training, and fostering a culture of ethical sensitivity are essential strategies for enhancing nurse well-being and patient care quality. Future research should continue to explore these dynamics and develop comprehensive strategies to support nurses in managing moral distress effectively.

In line with the current study, Mahdavi Feshtami et al. reported low levels of moral distress impact [28]. In the present study, “following unnecessary doctor’s orders” and “performing life-sustaining actions like resuscitation that only delay patient death” had the highest mean scores for the impact of moral distress. Sabri-Kouanchi et al. reported that situations causing the most moral distress include futile care to prolong death, unnecessary tests and treatments, and working with incompetent healthcare personnel [45]. Beheshtin et al. found that factors contributing to moral distress before and after COVID-19 were similar, with futile care and end-of-life issues being the main causes [46].

Differences in findings may be due to variations in research settings, samples, and data collection tools. Studies were conducted in different departments, cities, and time periods. Measurement tools for moral distress vary in terms of target population and specific items.

A significant correlation was found between the frequency of moral distress and age, work experience, and the number of children. Specifically, as age and the number of children increased, the frequency of moral distress decreased, while it increased with longer work experience. There was also a significant relationship between the intensity of moral distress and shift work, with nurses working rotating shifts experiencing higher intensity. Conversely, a significant inverse relationship was found between the impact of moral distress and both the number of children and work experience, with increased numbers of children and work experience reducing the impact of moral distress.

Similar results were observed in Brandi Showalter et al.‘s study, where an inverse correlation between moral distress and age was reported [47]. Sadeghi et al. and Rahmanian et al. also found a significant inverse relationship between age and the frequency of moral distress [44, 48].

Contrary to the present study, Mohammadi et al. found a significant relationship between the department and moral distress [43]. Sadeghi et al. found an inverse relationship between moral distress and work experience, with higher frequency of moral distress reported in emergency and psychiatric nurses, and higher intensity in emergency, orthopedic, and ICU nurses [48]. Mahdavi Feshtami et al. reported a positive relationship between the number of nurses and beds in a ward and the three dimensions of moral distress [28].

There are varying findings on the relationship between demographic variables and moral distress. It is unclear whether moral distress intensifies over time or diminishes with experience. There may be significant differences in the experience of moral distress depending on the environment and patient population [42]. In Petersen and Melzer’s study among German home care nurses, no demographic variables were associated with the level of disturbance caused by moral distress [49], suggesting the possibility of differences in study focuses—our study examined the frequency and intensity of moral distress rather than its consequences.

The clinical care quality score and its relationship with demographic characteristics indicated that clinical care quality was generally satisfactory among most nurses, with the highest quality reported in the communication dimension. Amiri et al. similarly reported the highest care quality in the communication dimension and the lowest in the physical dimension [40]. Gaalan et al., in their study in Mongolia, reported an overall satisfactory level of nursing care quality [50], and Mert Boğa et al. found high levels of perceived care quality among nurses [2].

In contrast, Moghaddam et al. reported the highest nurse performance in the dimensions of safe nursing care and physical safety [39]. Khorani et al. found that most nurses rated the quality of care as satisfactory, with the highest quality in the physical dimension and the lowest in the psychosocial dimension [34]. No significant relationship was found between any demographic variables and clinical care quality or its dimensions.

Bostani et al. and Heydari et al. similarly reported no significant relationship between demographic variables and nursing care quality [35, 51]. However, Khorani et al. found that female nurses provided higher quality care than males, and nurses with lower economic status reported lower care quality compared to those with a moderate economic status. In Khorani et al.‘s study, ethical sensitivity, gender, and economic status were the most significant predictors of nursing care quality [34]. Derzi-Ramandi et al. found higher care quality among female nurses, married individuals, and nurses with a master’s degree compared to those with a bachelor’s degree, with a direct relationship between work experience and care quality [52].

Gholami et al. found that among demographic variables, only age had a significant negative impact on care quality [53]. Given the diverse settings and cultures in which these studies were conducted, such varied results are not unexpected. Nonetheless, the necessity for further complementary studies on this important issue is evident.

### Limitations

Data collection was based on self-reporting. Therefore, given the workload and additional responsibilities of the nurses at the time of responding to the questions, caution should be exercised in generalizing the results. Potential reporting biases should also be considered, as nurses may not accurately convey their experiences or fully understand the psychological and professional impacts they face.

## Conclusion

Nurses who experience higher frequencies and intensities of moral distress tend to perform better in providing care within the scope of their practice, where no support from the system or physician orders is required. This is particularly evident in the physical aspect of care, where nurses can directly and immediately contribute to improving patient conditions.

### Recommendations

Given the complex nature of the phenomenon under discussion, it is recommended that qualitative studies be conducted to elucidate the factors contributing to moral distress and determine to what extent a nurse can deliver optimal performance despite experiencing moral distress. Additionally, studies that consider the three variables of moral distress, moral sensitivity, and quality of clinical care in various public and private centers with diverse demographic characteristics of nurses are suggested. This approach can provide a better understanding of how these variables interact and influence clinical care quality.

## Data Availability

The datasets used and/or analyzed during the current study are available from the corresponding author on reasonable request.

## Abbreviations

MDS-R: Revised Moral Distress Scale
QUALPAC: Quality Patient Care Scale
SD: Standard deviation
χ2: Chi square
